# Longitudinal decline in striatal dopamine transporter binding in Parkinson’s disease: associations with apathy and anhedonia

**DOI:** 10.1136/jnnp-2022-330790

**Published:** 2023-05-23

**Authors:** Harry Costello, Yumeya Yamamori, Suzanne Reeves, Anette-Eleonore Schrag, Robert Howard, Jonathan P Roiser

**Affiliations:** 1 Institute of Cognitive Neuroscience, University College London, London, UK; 2 Division of Psychiatry, University College London, London, UK; 3 UCL Institute of Neurology, University College London, London, UK

**Keywords:** APATHY, PARKINSON'S DISEASE, NEUROPSYCHIATRY, FUNCTIONAL IMAGING, DEPRESSION

## Abstract

**Background:**

Motivational symptoms such as apathy and anhedonia are common in Parkinson’s disease (PD), respond poorly to treatment, and are hypothesised to share underlying neural mechanisms. Striatal dopaminergic dysfunction is considered central to motivational symptoms in PD but the association has never been examined longitudinally. We investigated whether progression of dopaminergic dysfunction was associated with emergent apathy and anhedonia symptoms in PD.

**Methods:**

Longitudinal cohort study of 412 newly diagnosed patients with PD followed over 5 years as part of the Parkinson’s Progression Markers Initiative cohort.

Apathy and anhedonia were measured using a composite score derived from relevant items of the 15-item Geriatric Depression Scale (GDS-15) and part I of the MDS-Unified Parkinson’s Disease Rating Scale. Dopaminergic neurodegeneration was measured using repeated striatal dopamine transporter (DAT) imaging.

**Results:**

Linear mixed-effects modelling across all contemporaneous data points identified a significant negative relationship between striatal DAT specific binding ratio (SBR) and apathy/anhedonia symptoms, which emerged as PD progressed (interaction:β=−0.09, 95% CI (−0.15 to -0.03), p=0.002). Appearance and subsequent worsening of apathy/anhedonia symptoms began on average 2 years after diagnosis and below a threshold striatal DAT SBR level. The interaction between striatal DAT SBR and time was specific to apathy/anhedonia symptoms, with no evidence of a similar interaction for general depressive symptoms from the GDS-15 (excluding apathy/anhedonia items) (β=−0.06, 95% CI (−0.13 to 0.01)) or motor symptoms (β=0.20, 95% CI (−0.25 to 0.65)).

**Conclusions:**

Our findings support a central role for dopaminergic dysfunction in motivational symptoms in PD. Striatal DAT imaging may be a useful indicator of apathy/anhedonia risk that could inform intervention strategies.

**WHAT IS ALREADY KNOWN ON THIS TOPIC**

- Disorders of motivation, such as anhedonia and apathy, are common, disabling and respond poorly to treatment in Parkinson’s disease (PD). Striatal dopaminergic dysfunction is considered central to motivational symptoms, but this has never been examined longitudinally in PD.

**WHAT THIS STUDY ADDS**

- Using repeated striatal dopamine transporter (DAT) imaging over 5 years in a large cohort of patients with PD, we identified a robust negative relationship between striatal DAT binding and apathy/anhedonia symptoms in PD that emerged over time. As dopaminergic neurodegeneration progresses and striatal DAT declines, a threshold is reached beyond which apathy and anhedonia symptom burden begins to increase.

**HOW THIS STUDY MIGHT AFFECT RESEARCH, PRACTICE OR POLICY**

- Our findings provide mechanistic insights into motivational symptoms and suggest that striatal DAT imaging is a potentially useful marker of the risk of developing apathy and anhedonia in PD.

## Introduction

Characterised by deposition of α-synuclein in neurons and dopaminergic neuronal death, Parkinson’s disease (PD) is a model of dopamine dysfunction.^1^ Since its first description over 200 years ago, PD has been conceptualised as a movement disorder.^2 3^ However, it is now known that non-motor neuropsychiatric symptoms are common, and these have a greater negative impact on health-related quality of life than motor symptoms.^4 5^

Depression is a clinically and mechanistically heterogeneous syndrome, which has led efforts to define subtypes based on symptom profile.^6^ Motivational symptoms including apathy and anhedonia contribute to an ‘interest-activity’ cluster of depressive symptoms that predicts worse response to antidepressants in non-PD depression.^7^ Apathy and anhedonia are particularly common in PD, estimated to affect 40%^8^ and 46%^9^ of patients, respectively. Both are syndromes of motivation: apathy is defined as diminished initiation of and engagement in activities, while anhedonia, though originally conceptualised solely as an inability to experience pleasure, is now recognised to incorporate a loss of interest (ie, motivation) to act in order to seek pleasure.^10^ This key motivational element is supported by evidence that depressed patients with marked anhedonia retain hedonic capacity.^11^ Treatments for apathy and anhedonia are limited, and the presence of either predicts greater illness severity and poorer quality of life in patients with both depression^12 13^ and PD.^14 15^

Recent research suggests that apathy and anhedonia overlap and share underlying neurobiological mechanisms related to reward processing.^10^ Human and animal studies have shown that dopaminergic transmission is crucial in reward processing, especially motivated behaviour.^16^ Off dopaminergic medication, patients with PD have greater reward processing deficits^17^ and report worse apathy^18^ and anhedonia.^19^ Double-blind randomised controlled trials (RCTs) of dopaminergic agonists have indicated potential therapeutic efficacy in treating apathy^20^ and depression^21^ in PD, though findings have been mixed^22^ and better powered trials are needed.

Consistent with a role for dopamine in motivational symptoms, the ventral striatum, which receives extensive dopaminergic innervation from the midbrain, has been implicated in apathy and anhedonia.^10^ Stroke lesions in the ventral striatum and caudate nucleus are associated with the development of apathy, while greater atrophy and lower metabolism in the ventral striatum have been associated with apathy in PD.^23 24^ Unmedicated depressed individuals exhibit attenuated striatal activation during reward processing,^25^ and a large longitudinal study of motivational processing in adolescents identified a robust inverse relationship between anhedonia and reward-related ventral striatal activation.^26^

Dopamine transporter (DAT) imaging is commonly used as a diagnostic tool in PD, as it is sensitive to degeneration of dopaminergic nigrostriatal pathways.^27^ Striatal DAT decreases in PD as the disease progresses, owing to a loss of presynaptic dopaminergic projections from the substantia nigra and ventral tegmental area. DAT imaging, using ioflupane (123-I) single photon emission CT (SPECT), is clinically useful in distinguishing neurodegenerative Parkinson’s syndromes from cases of essential tremor or drug-induced parkinsonism,^28^ but is a poor predictor of the progression of motor symptoms.^29^ Animal studies have shown that selective lesions of substantia nigra dopamine neurons can induce motivational deficits and affective impairments, without eliciting motor deficits.^30^ In patients with PD, some smaller cross-sectional studies have reported that both apathy and depression are associated with lower striatal DAT specific binding ratio (SBR), although findings are mixed.^22 31–33^ A 2015 meta-analysis based on SPECT studies showed unchanged DAT binding in depression without PD,^34^ though more recent studies have reported lower DAT SBR in larger samples.^35^ However, it remains unclear whether motivational symptoms of depression are specifically driving this association. Therefore, we conducted the first study to examine the longitudinal relationship between striatal DAT SBR and the emergence and progression of apathy and anhedonia in PD.

We used data from the Parkinson’s Progression Markers Initiative (PPMI) cohort, an international multicentre cohort study. We tested the hypothesis that apathy and anhedonia symptoms in PD are driven by dopaminergic degeneration, indexed by DAT binding in the striatum. We predicted a negative relationship between striatal DAT binding and apathy and anhedonia, and that any such relationship would be specific to these symptoms, and not observed with general depressive symptoms.

## Methods

### Participants

All data were obtained from the PPMI database (https://www.ppmi-info.org/), first accessed July 2021. Launched in 2010 to identify markers of Parkinson’s onset and progression, PPMI includes repeated clinical measures and imaging biomarkers including (123-I)-SPECT.

PPMI enrolled untreated, newly diagnosed Parkinson’s patients and age-matched and sex-matched healthy controls, all of whom gave informed consent to participate prior to taking part (ClinicalTrials.gov NCT01141023). Participants underwent a standard battery of assessments^36^ including the MDS-Unified Parkinson’s Disease Rating Scale (MDS-UPDRS), the 15-item Geriatric Depression Scale (GDS) and DAT imaging with (123I)-SPECT repeatedly, over 5 years.

The current analysis included participants with a diagnosis of PD at baseline. In order to avoid capturing chronic symptoms in the context of premorbid depression prior to the development of PD, all participants who had received a diagnosis of major depressive disorder more than 5 years prior to diagnosis with PD were excluded (N=11).

### Apathy and anhedonia measure

We created a composite measure of apathy and anhedonia comprising items from the MDS-UPDRS and GDS-15 (table 1). The apathy/anhedonia score was operationalised based on the three-item ‘apathy/anhedonia’ factor of the GDS-15, which has been previously validated in older adults,^37^ and the apathy-specific measure in part I of the MDS-UPDRS.^38^

**Table 1:** Apathy/anhedonia composite measure

MDS-UPDRS part I, item 1.5:	Over the past week, have you felt indifferent to doing activities or being with people? Scale 0–4 (normal–severe)
GDS item 2:	Have you dropped many of your activities or interests? Scale yes (1)/no (0)
GDS item 9:	Do you prefer to stay at home, rather than going out and doing new things? Scale yes (1)/no (0)
GDS item 13:	Do you feel full of energy? Scale yes (0)/no (1)

GDS, Geriatric Depression Scale; MDS, Movement Disorder Society; UPDRS, Unified Parkinson’s Disease Rating Scale.

Parallel factor analysis of the apathy/anhedonia composite measure indicated a single factor loading (Cronbach’s alpha=0.60, 95% CI (0.56 to 0.62)).

A nine-item ‘depression’ factor from the GDS-15 (excluding the above apathy/anhedonia items) was used as a comparative measure to test whether any relationship with striatal DAT binding was specific to apathy/anhedonia.^39^ This ‘depression’ factor was identified in an independent cohort using factor analysis to investigate depressive symptom clusters in patients with PD, and comprises GDS-15 items 1, 3, 5, 7–8, 11–12 and 14–15.^39^

### Image processing and calculation of striatal DAT SBR by PPMI

All images were analysed according to the PPMI imaging technical operations manual and had undergone analysis to determine striatal DAT specific binding ratio (SBR) (see online supplemental methods for details).^40^ Regions of interest (ROIs) included the left and right caudate, the left and right putamen, and the occipital cortex (reference tissue). Count densities for each region were extracted and used to calculate the SBR for each of the striatal ROI. SBR is calculated as: (target region/reference region)–1. To account for asymmetry, the minimum SBR for each region was used. Striatal SBR was a summative measure of putamen and caudate SBR. Hemispheric side of measurement (left/right) was accounted for in analysis.

10.1136/jnnp-2022-330790.supp1 Supplementary data

### Statistical analysis

We used linear mixed-effects modelling to examine the relationship between apathy/anhedonia score (dependent variable) and striatal DAT SBR (independent variable), which were acquired contemporaneously, and how this relationship changed over the progression of illness. This involved fitting both the main effect of striatal DAT SBR (across all time points) and its interaction with time. This allowed interindividual heterogeneity and unequal follow-up intervals to be accommodated by incorporating random effects. Random intercept terms at the participant level were tested, with random slopes for time (defined as year of follow-up assessment). The interaction term between striatal DAT SBR and time allowed us to assess how the relationship between apathy/anhedonia and striatal DAT SBR changed over time, using all available data. When a significant interaction was identified, post hoc tests were conducted using Pearson’s correlations at each time point separately.

Two sets of regression were conducted: (1) unadjusted and (2) adjusted for age, sex, years of education, number of missing years and duration of PD (all at baseline); plus cognition (Montreal Cognitive Assessment), severity of motor symptoms (MDS-UPDRS part III score, ‘off’ medication), stage of disease/functional disability (Hoehn and Yahr scale), levodopa equivalent dose (LED), antidepressant medication status, hemispheric side of striatal DAT SBR and the GDS-15 ‘depression’ factor (all at each contemporaneous time point). Model fit was tested using the Akaike information criterion (AIC).

For LED calculation, the LED of each drug was calculated by multiplying its daily dose by its conversion factor, and total LED at a particular time point was then calculated by adding the LED of all the drugs. Further details on the collection of these data can be obtained from the PPMI website (https://www.ppmi-info.org/study-design).^41^

To test whether findings were specific to apathy/anhedonia, mixed-effects modelling was repeated with motor symptom severity and the GDS-15 ‘depression’ factor as dependent variables in separate analyses, incorporating apathy/anhedonia score as a covariate.

The ‘two-lines’ test (see online supplemental methods for summary) was performed to test the validity of a threshold effect of striatal DAT SBR on apathy/anhedonia.^42^

All statistical analyses were performed in R V.4.1.2. The R package ‘lme4’ was used for mixed effects modelling, and the ‘twolines’ package was used for the two-lines test.

## Results

### Participant characteristics

In total 412 participants with PD were included at baseline with dropout of one-quarter of participants by year 5 (table 2).

**Table 2:** Characteristics of PPMI participants with PD

	Baseline HC	Baseline PD	Year 1	Year 2	Year 3	Year 4	Year 5
n	196	412	384	367	355	335	304
Age at entry	60.82 (11.23)	61.77 (9.76)	61.66 (9.86)	61.66 (9.86)	61.64 (9.86)	61.40 (9.96)	60.94 (9.84)
Age at diagnosis	–	61.22 (9.73)	61.1 (9.83)	61.1 (9.84)	61.09 (9.82)	60.86 (9.92)	60.39 (9.83)
Duration of PD at cohort entry (years)	–	6.56 (6.39)	6.64 (6.51)	6.70 (6.56)	6.65 (6.54)	6.57 (6.48)	6.6 (6.53)
Years of education	16.04 (2.89)	15.54 (2.99)	15.47 (2.9)	15.6 (2.87)	15.61 (2.92)	15.62 (2.90)	15.61 (2.97)
Male (%)	126/196 (64)	273/412 (66)	255/384 (66)	244/367 (66)	235/355 (66)	225/335 (67)	205/304 (67)
Apathy/anhedonia composite	0.55 (0.87)	1.21 (1.14)	1.41 (1.30)	1.51 (1.43)	1.48 (1.48)	1.58 (1.49)	1.67 (1.54)
MDS-UPDRS III	1.21 (2.20)	20.93 (8.80)	25.25 (10.92)	27.62 (11.39)	29.58 (12.35)	30.97 (12.20)	31.27 (12.28)
UPDRS total	4.56 (4.40)	32.41 (13.14)	39.59 (16.12)	43.02 (16.97)	46.33 (18.80)	48.70 (19.68)	49.96 (19.04)
MOCA	28.23 (1.11)	27.13 (2.28)	26.28 (2.82)	26.27 (3.14)	26.37 (3.01)	26.42 (3.57)	26.53 (3.53)
GDS	1.29 (2.10)	2.29 (2.43)	2.53 (2.92)	2.58 (2.87)	2.58 (2.79)	2.59 (2.84)	2.78 (2.80)
GDS>5	9/196 (5%)	55/412 (13%)	61/384 (16%)	63/366 (17%)	58/355 (16%)	58/333 (17%)	61/303 (20%)
STAI	57.13 (14.09)	65.05 (18.14)	64.92 (18.63)	64.65 (18.33)	64.41 (18.38)	64.71 (18.71)	64.60 (18.95)
% commenced PD medication	–	0/412 (0)	225/382 (59)	310/367 (84)	329 (355) 92.7	319/332 (96.1)	293/304 (96.4)
% on antidepressant	29/196 (14.8)	29/412 (7.0)	29/384 (7.6)	33/367 (9.0)	30/355 (8.5)	32/335 (9.6)	26/278 (9.4)
No DAT scans	193	408	360	341	10	280	7
Striatal DAT SBR	4.987 (1.12)	2.50 (0.75)	2.21 (0.66)	2.07 (0.68)	1.67 (0.54)	1.82 (0.64)	1.30 (0.67)

Mean (SD); N (%).

DAT-SBR, dopamine transporter specific binding ratio; GDS, Geriatric Depression Scale; HC, healthy control; MDS-UPDRS, Movement Disorder Society-Unified Parkinson’s Disease Rating Scale; MOCA, Montreal Cognitive Assessment; PD, Parkinson’s disease; PPMI, Parkinson’s Progression Markers Initiative; STAI, State-Trait Anxiety Inventory.

**Figure 1:**
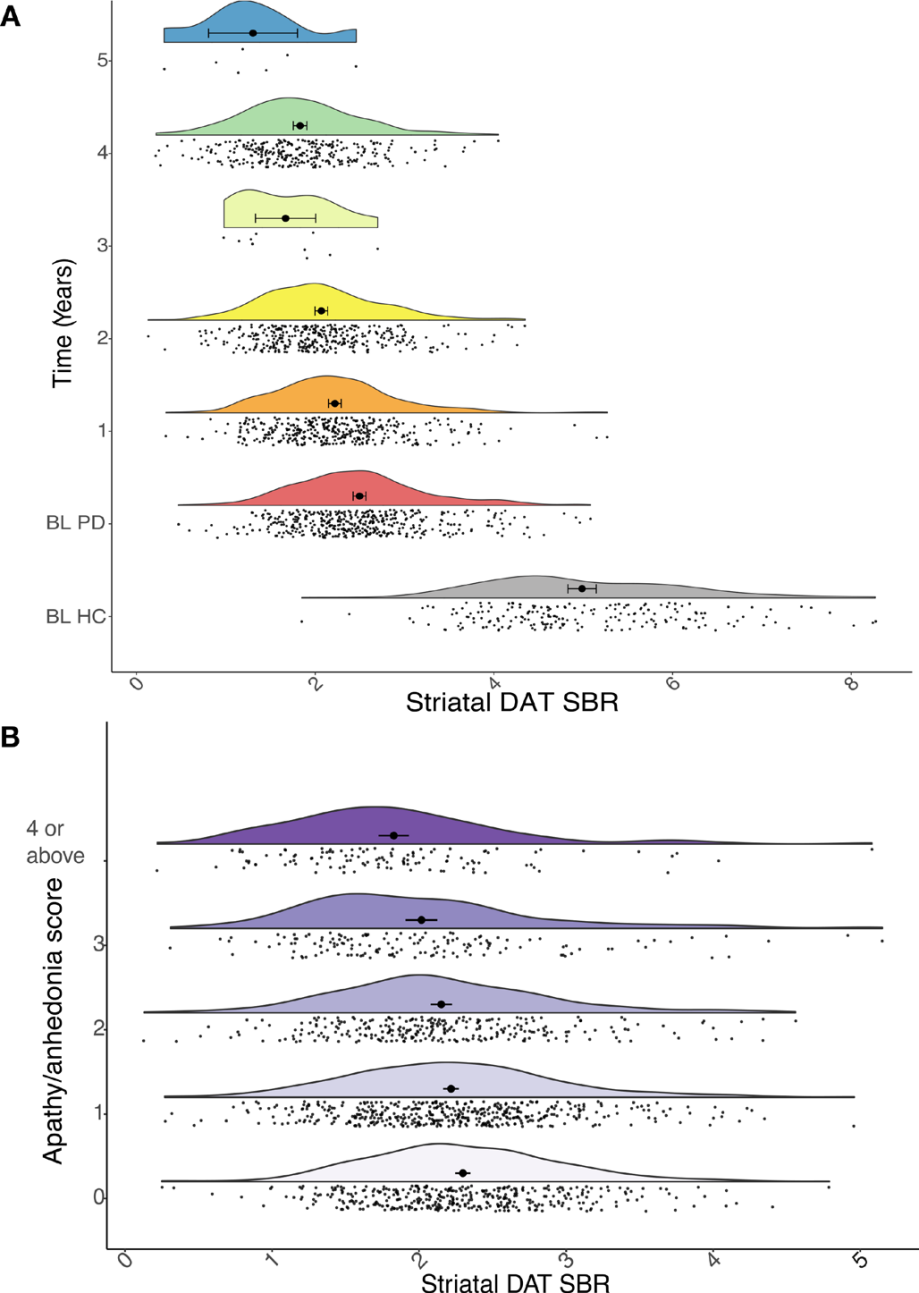
(A) Raincloud plots showing the expected progressive reduction in striatal dopamine transporter (DAT) specific binding ratio (SBR) in Parkinson’s disease (PD, coloured plots) over time relative to baseline (BL), compared with healthy controls (HC, grey plot) at BL. (B) Raincloud plots showing striatal DAT SBR at different levels of apathy/anhedonia, across all years.

Almost all (98.8%) DAT imaging was completed at baseline and in years 1, 2 and 4, with only 17 participants imaged in years 3 and 5. However, due to the mixed-effects modelling approach, we could incorporate all available data into the analysis. As expected, at baseline striatal DAT SBR in PD participants was on average around half that of healthy controls; and there was evidence of a marked decline over time (mean±SD percentage reduction from baseline: year 1=−9.7%±17.4%, year 2=−16.6%±17.7%, year 4=−26.6%±18.4%; figure 1A).

As anticipated, motor symptom severity increased over time (MDS-UPDRS III: β=4.0, 95% CI (3.7 to 4.3), p<0.001). Although all participants were unmedicated at baseline, a majority (59%) had commenced dopaminergic medication by year 1. One-quarter of participants (25%) with PD were taking antidepressant medication at baseline, but only 13% had a GDS-15 score >5 (suggestive of clinical depression) and indication for treatment was not available. Apathy/anhedonia composite score increased over time (β=0.22, 95% CI (0.10 to 0.34), p<0.001) (figure 1B).

### Relationship between striatal DAT SBR and apathy/anhedonia

**Figure 2:**
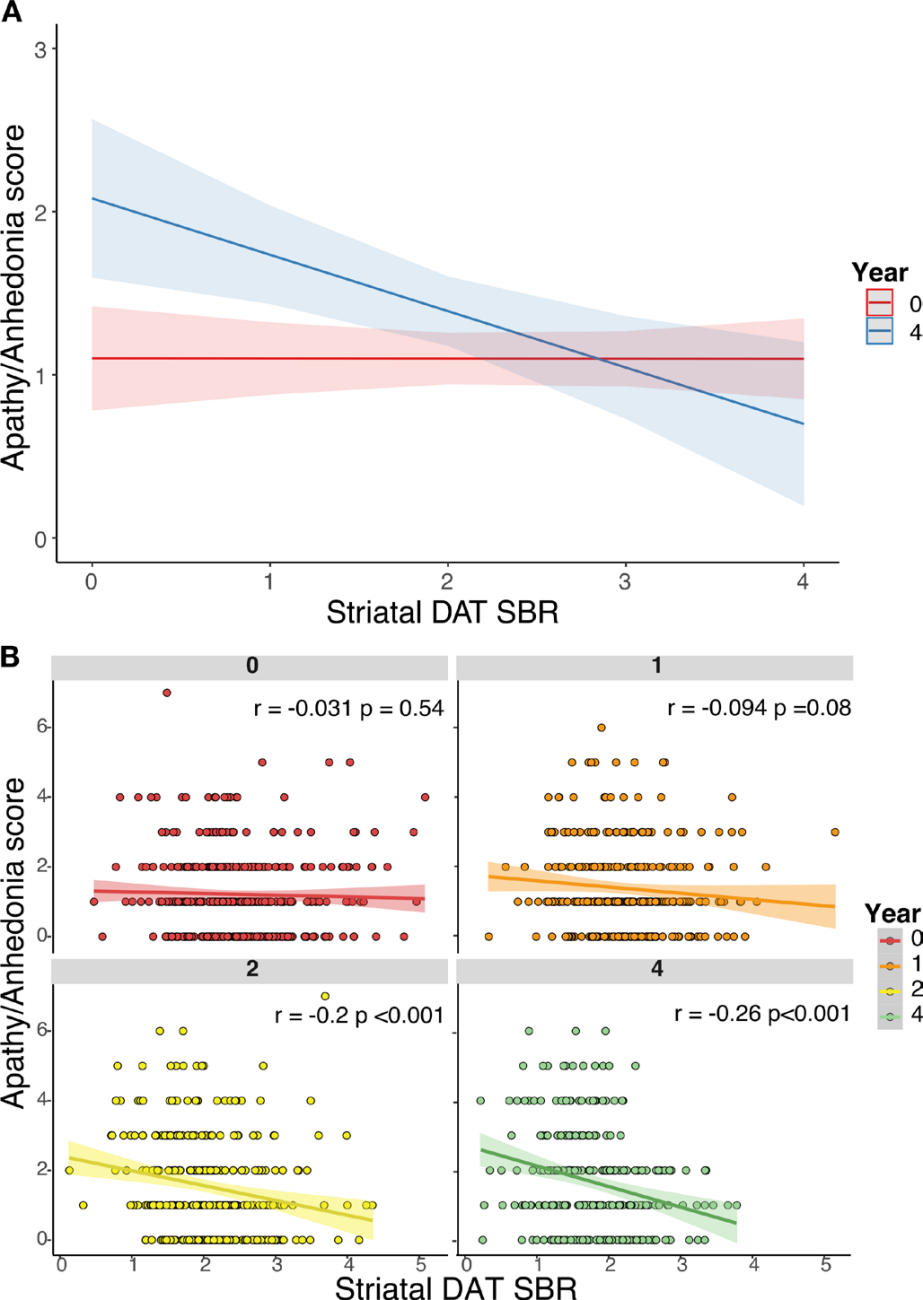
(A) Adjusted mixed-effects model simulation of predicted apathy/anhedonia score by striatal dopamine transporter (DAT) specific binding ratio (SBR) and time (years 0 and 4 simulated). (B) Scatter plots displaying unadjusted linear regressions between striatal DAT SBR and apathy/anhedonia score at years 0 (baseline), 1, 2 and 4, showing the strengthening of the relationship over time.

**Figure 3:**
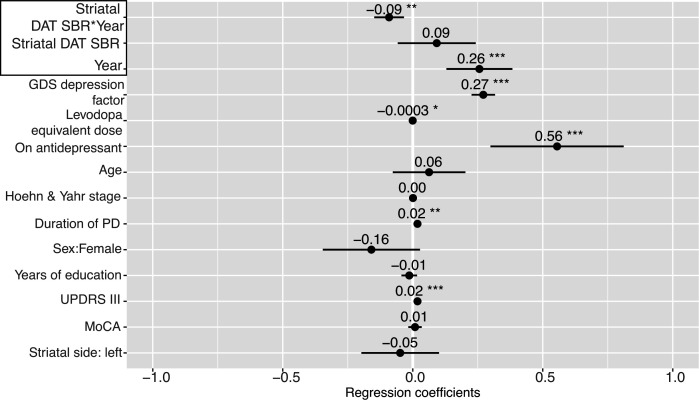
Mixed-effects model investigating the relationship between striatal dopamine transporter (DAT) specific binding ratio (SBR) and apathy/anhedonia longitudinally, adjusted for age, sex, years of education and duration of Parkinson’s disease (PD) (assessed at baseline only); as well as cognition (assessed with the Montreal Cognitive Assessment (MoCA)), severity of motor symptoms (MDS-UPDRS part III score), functional disability (Hoehn and Yahr scale), levodopa equivalent dose, antidepressant status, hemispheric side of striatal DAT SBR and the GDS-15 ‘depression’ factor (assessed contemporaneously). Points represent estimated regression coefficients and bars represent 95% CIs; *p<0.05, **p<0.01, ***p<0.001. GDS-15, 15-item Geriatric Depression Scale; MDS-UPDRS, Movement Disorder Society-Unified Parkinson’s Disease Rating Scale.

Longitudinal analysis revealed that, while the overall relationship between striatal DAT SBR and apathy/anhedonia across all time points was non-significant (β=0.09, 95% CI (−0.06 to 0.24), p=0.2), there was a significant interaction with time in the unadjusted model (β=−0.09, 95% CI (−0.15 to -0.03), p<0.001) (figure 2). A similar relationship was observed in the adjusted model (including: demographic factors, cognition, motor symptoms, LED, antidepressant status and the GDS-15 ‘depression’ factor, which excludes apathy/anhedonia items), which was more parsimonious (AIC: adjusted=3143.5, unadjusted=3762.1) (figures 2, 3A). Post hoc analysis revealed that striatal DAT SBR was not significantly associated with apathy/anhedonia at baseline (r=0.03, p=0.54), but this relationship emerged over follow-up (year 4: r=−0.26, p<0.01), strengthening as time progressed. In other words, as striatal DAT SBR decreases and apathy/anhedonia increases over the course of disease progression, a negative relationship between the two develops (figure 2B). Similar results were observed in separate analyses of the caudate and putamen striatal subdivisions (online supplemental figure S1A,B).

This interaction between striatal DAT SBR and time was only observed for apathy/anhedonia. Separate sensitivity analyses using MDS-UPDRS part III score (online supplemental figure S2A) and the GDS-15 ‘depression’ factor (online supplemental figure S2B) as dependent variables, using an adjusted model including apathy/anhedonia as a covariate, showed no significant interactions with time. Though motor symptoms increased over time and lower striatal DAT SBR was associated with worse motor symptoms across all time points (main effect: β=−1.30, 95% CI (−2.4 to -0.22), p=0.02) (online supplemental figure S2A), unlike apathy/anhedonia, this association did not strengthen over time (interaction: β=0.20, 95% CI (−0.25 to 0.65), p=0.4) (online supplemental figure S2A). We found no significant relationship between striatal DAT SBR and the GDS-15 ‘depression’ factor (main effect across all time points: β=−0.06, 95% CI (−0.14 to 0.26), p=0.5; interaction with time: β=−0.06, 95% CI (−0.13 to 0.01), p=0.09) (online supplemental figure S2B).

To investigate clinical relevance of the association between apathy/anhedonia and striatal DAT SBR, we analysed the three items of the GDS contributing to our apathy/anhedonia composite measure as the dependent variable, which has previously been validated as a clinical measure of apathy (GDS-3A).^37^ A GDS-3A score of ≥2 has high specificity for clinically relevant apathy in older adults, equivalent to a score of ≥14 using the Starkstein apathy scale.^37^ In adjusted analyses, similar results were observed for both continuous and categorical analyses of the GDS-3A, with significant interactions between striatal DAT SBR and time (online supplemental figure S3A,B).

### Is the association between striatal DAT SBR and apathy/anhedonia dependent on change from baseline?

Restricting analysis to postbaseline time points, we incorporated baseline DAT SBR as a covariate within the adjusted model (online supplemental figure S4,S5), and also examined change from baseline DAT SBR, in separate analyses (online supplemental results). There was no evidence that baseline DAT SBR predicted later apathy/anhedonia, and no evidence for an association with change-from-baseline DAT SBR (online supplemental figure S4, top); this is in contrast to the clear strengthening of association between absolute striatal DAT SBR and apathy/anhedonia score as PD progresses (online supplemental figure S4, bottom).

These analyses suggest that it is the absolute value of striatal DAT SBR, and not change from baseline, that is associated with apathy/anhedonia.

### Exploratory analysis of threshold striatal DAT SBR in developing apathy/anhedonia

Using the two-lines test,^42^ we assessed whether a threshold striatal DAT SBR value exists, below which the relationship with apathy/anhedonia begins to emerge.

**Figure 4:**
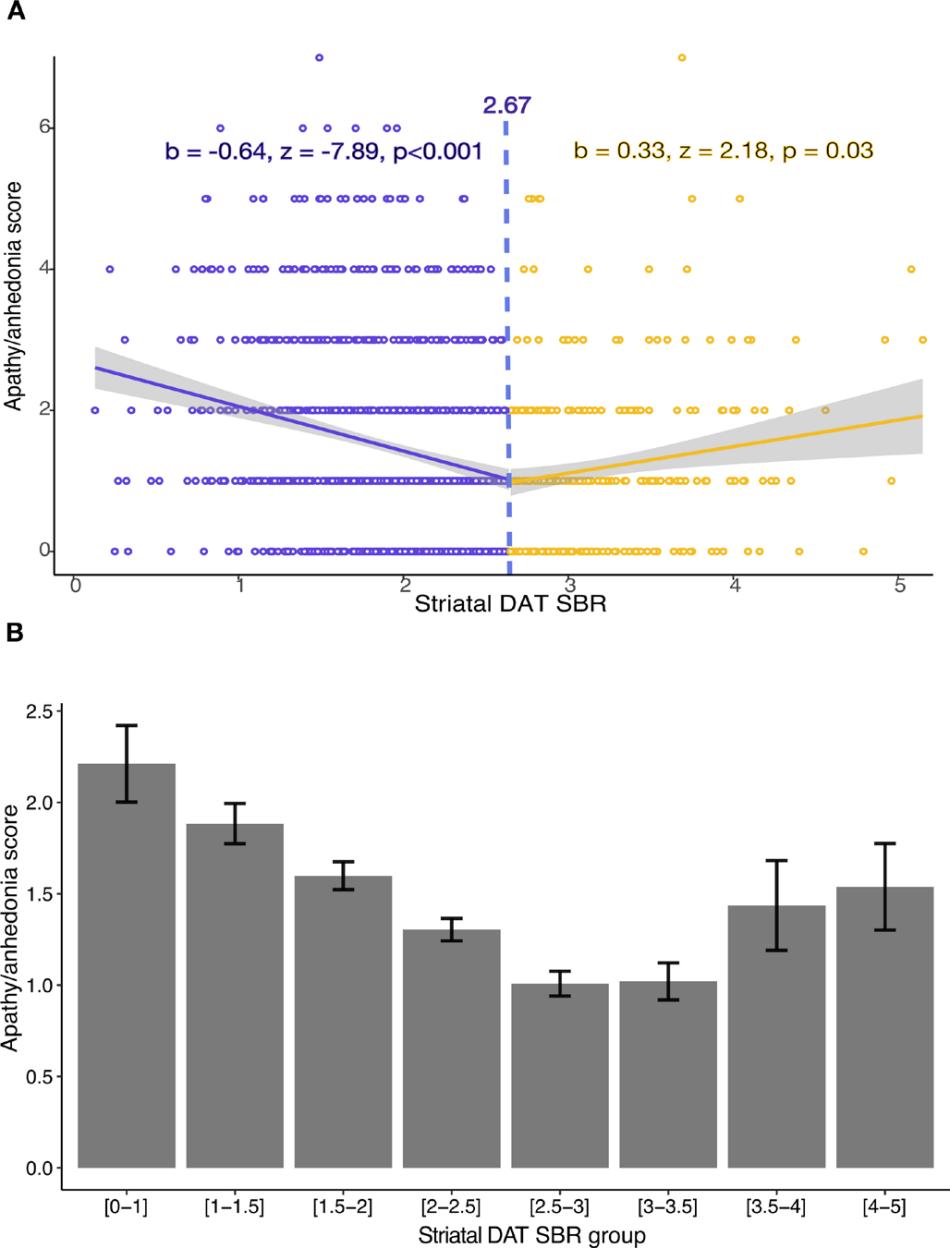
(A) Two-lines test assessing the validity of a threshold effect between striatal dopamine transporter (DAT) specific binding ratio (SBR) and apathy/anhedonia score, across all available contemporaneously acquired data points and years of follow-up. The two-lines test estimates a DAT SBR threshold of ~2.7, beyond which apathy/anhedonia starts to increase. (B) Mean apathy/anhedonia score and SE at binned increments of SBR, across all available contemporaneously acquired data points and years of follow-up.

Analysis including all available contemporaneously acquired data points, across all years, revealed the threshold effect, consistent with a non-linear relationship (figure 4). Both the upward (p=0.03) and downward (p<0.001) regression slopes achieved statistical significance (figure 4). A threshold striatal DAT SBR value of ~2.67 was estimated, below which a negative relationship with apathy and anhedonia develops. This is consistent with an explanation according to which apathy/anhedonia symptoms start to increase beyond a certain level of dopaminergic degeneration. However, we note that the Robin-Hood algorithm used in the two-lines test primarily tests for the presence of a threshold and cannot precisely identify the precise turning point value. Therefore, the significant upward regression slope is conceivably an artefact of the operation of the algorithm, rather than evidence of patients with the highest striatal SAT SBR having higher apathy/anhedonia scores, which was not evident in other analyses (figure 2B).

## Discussion

This is the first longitudinal analysis of the relationship between striatal dopaminergic dysfunction and apathy and anhedonia in PD. Using a common clinically used neuroimaging marker, we found that a negative relationship between striatal DAT SBR and apathy/anhedonia symptoms emerged beyond a certain threshold of dopaminergic neurodegeneration. This relationship is not present during the early stages of illness, but develops over time as PD pathology progresses and a threshold level of pre-synaptic striatal dopaminergic neurodegeneration is crossed.

Our findings support existing evidence for the important role striatal dopamine dysfunction plays in disorders of motivation^10 23^ and suggests that striatal DAT imaging could contribute to indicating the risk of developing motivational symptoms in PD. In patients whose striatal DAT SBR is particularly low, there is a scope to monitor apathy and anhedonia symptoms, or to intervene early with interventions such as behavioural activation therapy which has been shown to be potentially effective in treating these symptoms in PD.^43^ However, further validation of striatal DAT SBR as a potential marker of apathy/anhedonia risk is needed, especially as the Robin-Hood algorithm used in the two-lines test primarily tests for the presence of a threshold effect and cannot precisely identify the true turning point value.

In contrast to the pattern, we observed in relation to apathy and anhedonia, striatal DAT SBR showed a consistent relationship with motor symptoms over time, with no evidence for an interaction. This aligns with previous studies,^29^ and may reflect differential degeneration of functional subdivisions of the striatum. At diagnosis with PD, it is likely that up to 80% of dopaminergic projections to the caudate and putamen have already been lost.^44 45^ Dopaminergic denervation of the caudal-motor subregion of the striatum, which receives input from the primary motor and premotor cortices, is central to the development of motor symptoms.^46^ Owing to the degree of early denervation in the caudal-motor region, a floor effect might explain why the relationship between striatal DAT SBR and motor symptoms does not emerge over time following diagnosis.

The association between striatal DAT SBR and apathy/anhedonia symptoms that emerges over time was present in putamen and caudate striatal subregions; however, the PPMI dataset does not include ventral striatum DAT binding. The ventral striatum, which receives dopaminergic input from the ventral tegmental area, is relatively spared in early PD^45 46^ and is believed to play a crucial role in motivated behaviour. Atrophy in this region has been linked with apathy across neurodegenerative disorders.^23^ We speculate that the interaction we observed with time may occur as a consequence of the later dopaminergic denervation of the ventral striatum, with motivational symptoms being less affected by the earlier caudal-motor striatal denervation.

A recent paper, also using the PPMI database, investigated DAT SBR in patients with PD who reported no apathy versus those who scored ≥1 on the apathy item of the MDS-UPDRS part I.^46^ Lower DAT SBR was found in the apathetic PD group in several extrastriatal regions, including the orbitofrontal cortex and posterior cingulate cortex.^46^ In conjunction with the results presented here, this supports existing evidence that dopaminergic modulation of frontostriatal circuitry plays a crucial role in amotivational symptoms in PD.^47 48^ However, this study only analysed DAT binding at baseline and one year following diagnosis, which may explain why they failed to detect any differences in the striatum (which is consistent with our own findings).^46^ Our study is the first to investigate individual longitudinal changes in striatal DAT binding beyond the first year following diagnosis and its relationship with motivational symptoms, identifying a threshold effect beyond which apathy and anhedonia symptom burden begins to increase. Importantly, we find that the relationship between striatal DAT SBR and apathy/anhedonia is only evident beyond the first year following diagnosis.

The function of dopamine in the striatum has been proposed to depend on phasic and tonic dopaminergic cell firing modes.^49 50^ Short-latency phasic firing of dopaminergic neurons in the striatum encodes reward prediction errors, crucial for reinforcement learning, while tonic levels of activity are thought to signal average reward valuation.^48^ Animal studies have shown that different striatal regions receive distinct dopamine signals encoding different aspects of motivational stimuli and their prediction.^51^

A gradient in the rate of reuptake of dopamine, from ventral to dorsal regions of the striatum, has been reported.^52^ This is consistent with the notion that that dorsal striatal regions are more sensitive to phasic signalling while the ventral striatum has greater utilisation of tonic signalling, which has been proposed to represent reward valuation.^48 52^ The pattern and timing of neurodegeneration in PD, which progresses from dorsal to ventral striatum, may explain why apathy/anhedonia symptoms occur later in disease, and supports existing evidence that anhedonia and apathy are a consequence of how reward valuation is represented by dopaminergic signalling.^53^

Though our findings are specific to PD, they may also reveal mechanistic insights into apathy and anhedonia transdiagnostically. Individual variation in the basal tone of different dopaminergic projection systems is hypothesised to be crucial in cognitive biases and susceptibility to psychiatric symptoms such as apathy and anhedonia.^48^ However, DAT imaging in depression has been conducted only in small samples, with conflicting findings.^54^ Future studies are required to further explore the relationship between striatal dopaminergic dysfunction and the emergence of specific neuropsychiatric symptoms. Additionally, the role of dopaminergic medication in the treatment of apathy and anhedonia requires further investigation. Methylphenidate, a noradrenaline-dopamine reuptake inhibitor, has recently been found to have efficacy in treatment of apathy in Alzheimer’s dementia.^55^ Further understanding of the effects of dopaminergic medication regime on apathy and anhedonia in PD is needed, in addition to RCTs of dopaminergic agents for apathy and anhedonia across different disorders.

## Limitations

The PPMI cohort only includes recently diagnosed patients with PD and may not be applicable to individuals in the later stages of the condition where the spread of neurodegeneration and systems involved are likely more complex.

Though our composite measure of apathy and anhedonia exclusively incorporated items specifically designed to measure these symptoms, it has not been validated previously, and it is unclear from this measure alone whether participants developed clinically meaningful levels of apathy and anhedonia during the study. However, we addressed this by additionally examining a measure that has been clinically validated (the GDS-3A,^37^ which formed the bulk of our apathy/anhedonia composite measure). This showed results consistent with our primary analyses, with clear interactions with time using both continuous and categorical apathy measures. However, we note that the GDS-3A has low sensitivity for apathy, meaning that some clinically relevant cases will be missed by this measure. It would have been preferable to have used validated measures of apathy and anhedonia, and to have assessed whether these symptoms were clinically relevant, but unfortunately this information is not available in the PPMI dataset.

The associations we observed between apathy and anhedonia and striatal DAT SBR potentially could be a consequence of other Parkinson’s symptoms, functional disability or depression. However, this is unlikely as all models were adjusted for cognition, motor symptoms, disease duration, functional disability and general depressive symptoms. Additionally, our findings were relatively specific to apathy/anhedonia with no significant relationship observed between striatal DAT SBR and general depressive symptoms in sensitivity analyses; although we note that the upper end of the 95% CI for general depression scores was near zero, and therefore, it is possible that a weak interaction exists which we did not have statistical power to detect, even in the large PPMI dataset.

Although DAT imaging is used clinically as a sensitive measure of presynaptic dopaminergic neurodegeneration in PD,^56 57^ the radioligand (123I)-ioflupane used in the PPMI cohort has also been suggested to have a modest affinity for the serotonin transporter.^58^ The degree to which DAT imaging reflects solely dopaminergic function is therefore uncertain. However, it is known that individual neurons can release multiple transmitters from the same synapse^59^ and recent research suggests co-release of dopamine and serotonin is functionally relevant,^60^ although co-localisation of different neurotransmitter transporters is sparse.^61^ As a consequence, it is likely that the interplay between dopamine and serotonin in the striatum is important in motivational symptoms, as suggested by animal studies.^48 49^ LED and antidepressant medication status were also incorporated into our modelling, with no effect on the interaction between DAT SBR and time.

Finally, although healthy controls underwent SPECT imaging at baseline and had average striatal DAT SBR levels almost double that of Parkinson’s participants, they did not have repeat imaging, so comparison to a control group longitudinally was not possible.

## Conclusion

In PD, striatal DAT SBR is associated with apathy and anhedonia symptoms over time. As dopaminergic neurodegeneration progresses and striatal DAT SBR declines, a threshold is reached beyond which apathy and anhedonia symptom burden begins to increase. This effect is relatively specific to apathy and anhedonia, with no such interaction evident for depressive or motor symptoms, and is consistent with prior evidence of the crucial role of striatal dopaminergic dysfunction in motivational dysfunction. Further validation of striatal DAT imaging as a potential clinically useful marker of apathy and anhedonia risk in PD is warranted.

## Data Availability

Data are available on reasonable request. PPMI is an open access dataset which is available on request from (https://www.ppmi-info.org/access-data-specimens/download-data). All code for this study is available on reasonable request.
