# Supplementary material for: Longitudinal decline in striatal dopamine transporter binding in Parkinson’s disease: associations with apathy and anhedonia
Source: J Neurol Neurosurg Psychiatry. 2023 May 23;94(10):863–70. doi: 10.1136/jnnp-2022-330790 (PMC10511995; doi:10.1136/jnnp-2022-330790)
Supplement: Supplementary data [file jnnp-2022-330790supp001.pdf]

**Supplement**

**Summary of the 'two-lines' test**

The two-lines test, which relies on using two regression lines (one for low values of the predictor, the other for high values), has been proposed as a more effective and valid method than quadratic regression modelling to indicate a non-linear relationship. Using this method also allows identification of a "breakpoint" value, using the "Robin Hood" algorithm. The breakpoint value is identified by reallocating observations from the statistically stronger of the two lines to the weaker, increasing statistical power to detect the inverse unimodal shape. For an association to qualify as a U-shaped using the two-lines test, the two regression lines must differ in direction in addition to being independently significant.[37] However, it should be noted that the threshold value identified by the Robin Hood algorithm is not directly interpretable, as the break point is identified from a range of candidate values, meaning that it is not estimated precisely.

**Figure S1.** Mixed-effects model investigating the relationship between dopamine transporter (DAT) specific binding ratio (SBR) and apathy/anhedonia in different striatal subdivisions longitudinally**. A.** Caudate. **B.** Putamen. Points represent estimated regression coefficients and bars represent 95% confidence intervals; p<0.05*, p<0.01**, p<0.001***.

**A.**

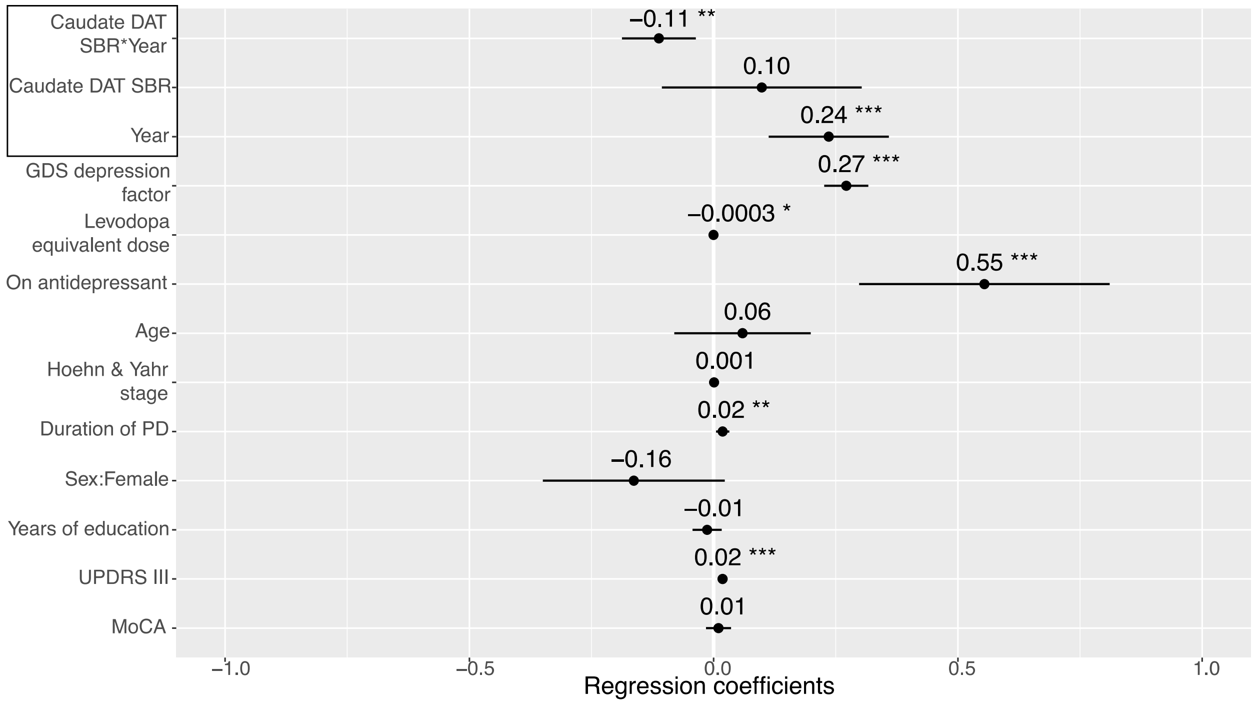


**B.**

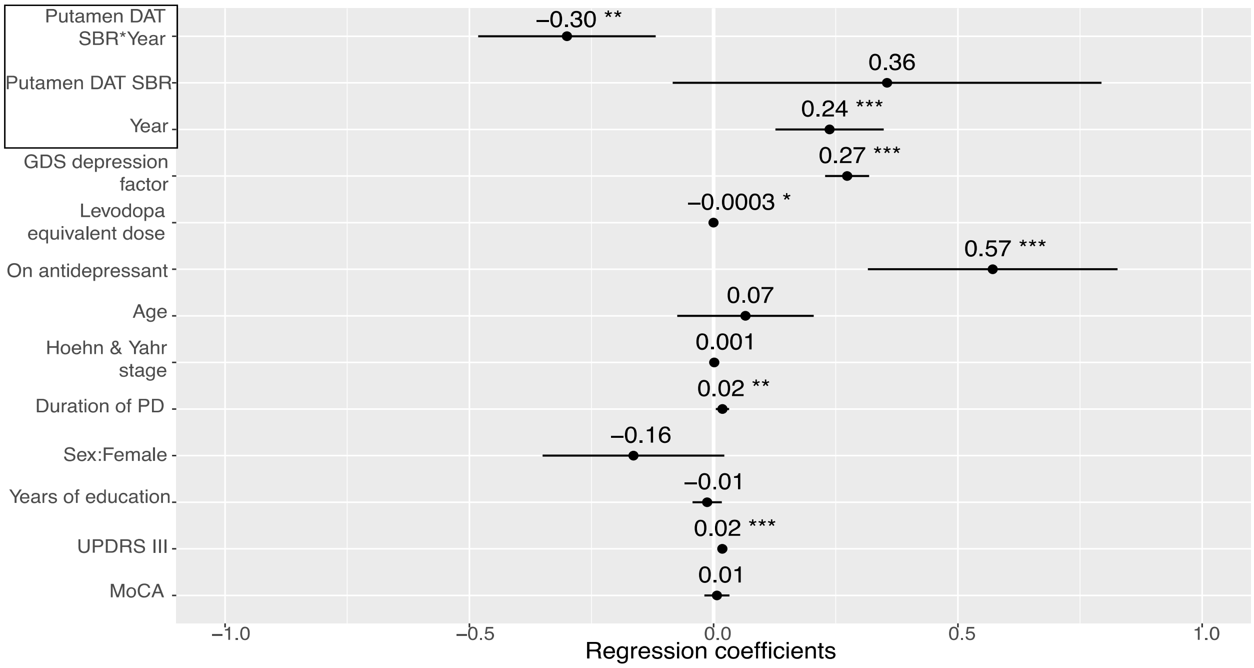


Analysis of the association between apathy/anhedonia and DAT SBR in different striatal subdivisions found similar interactions with time in both the caudate ((β=-0.11, 95%CI [-0.19 -0.04], p<0.001) and putamen (β=-0.30, 95%CI [-0.48 -0.12], p<0.001; Figure S1).

**Figure S2**. Sensitivity analyses examining the relationship between dopamine transporter (DAT) specific binding ratio (SBR) and motor and depression symptoms, using separate mixed-effects models. Apathy/anhedonia score has been replaced as the dependent variable with (**A**) MDS-UPDRS part III score and (**B**) the Geriatric Depression Scale-15 (GDS) “depression” factor. Points represent estimated regression coefficients and bars represent 95% confidence intervals; p<0.05*, p<0.01**, p<0.001***. The interaction between striatal dopamine transporter specific binding ratio (SBR) and time was non-significant for both models.

**A.**

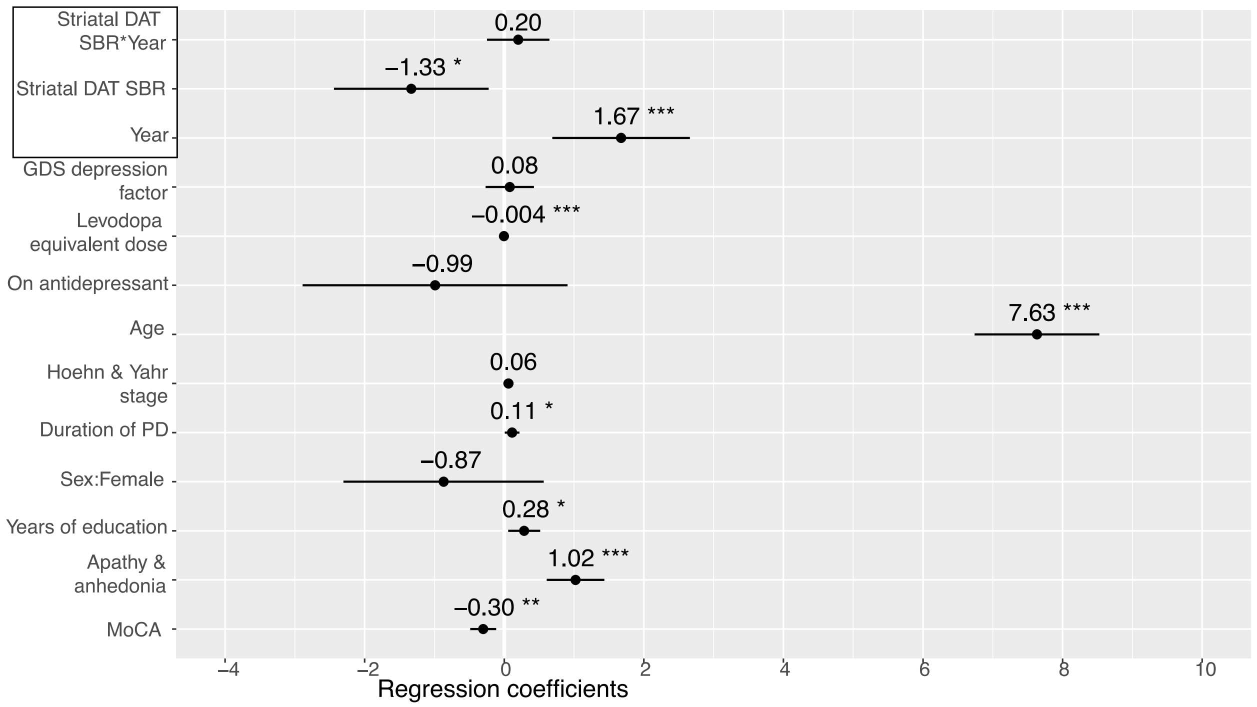


**B.**

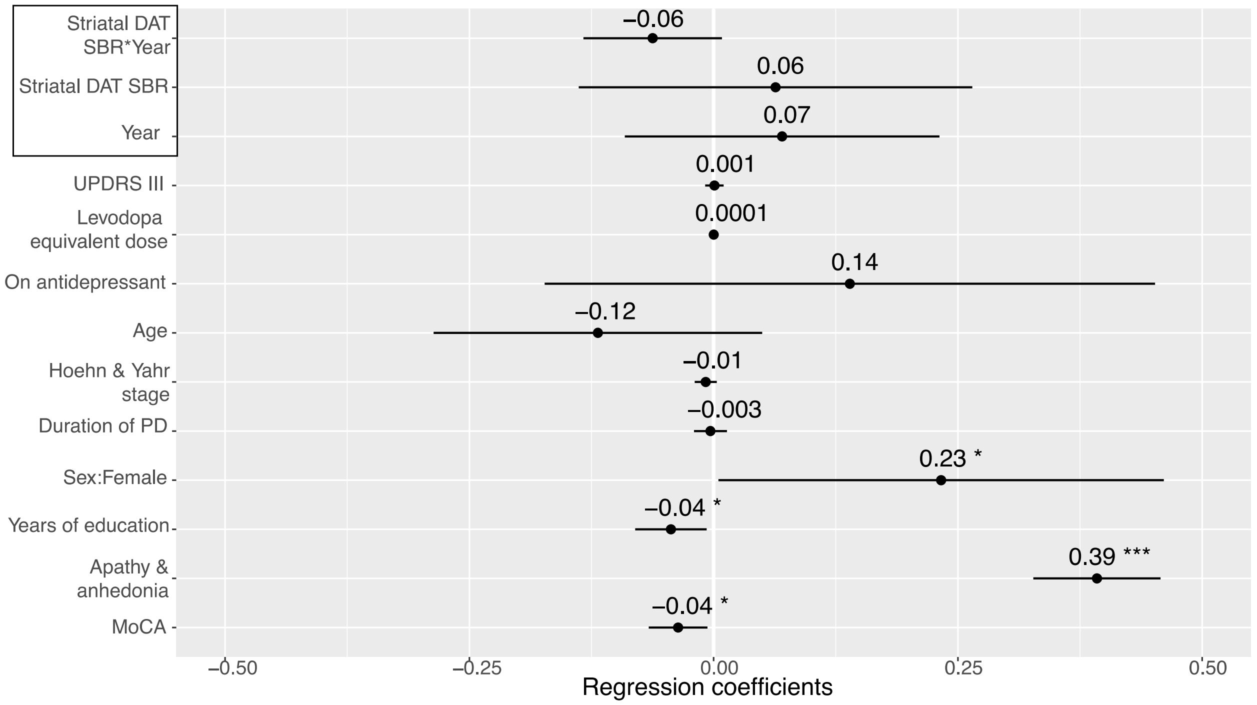

**Figure S3. A. Linear mixed effects model** investigating the relationship between striatal dopamine transporter (DAT) specific binding ratio (SBR) and the Geriatric Depression Scale (GDS)-3A, a three-item subset of the GDA-15, previously validated as a clinical measure of apathy. **B. Logistic mixed effects model** investigating the relationship between striatal DAT SBR and a categorical outcome using a cut-off of ≥2 on the GDS-3A that has high specificity for clinically relevant apathy.

**A.**

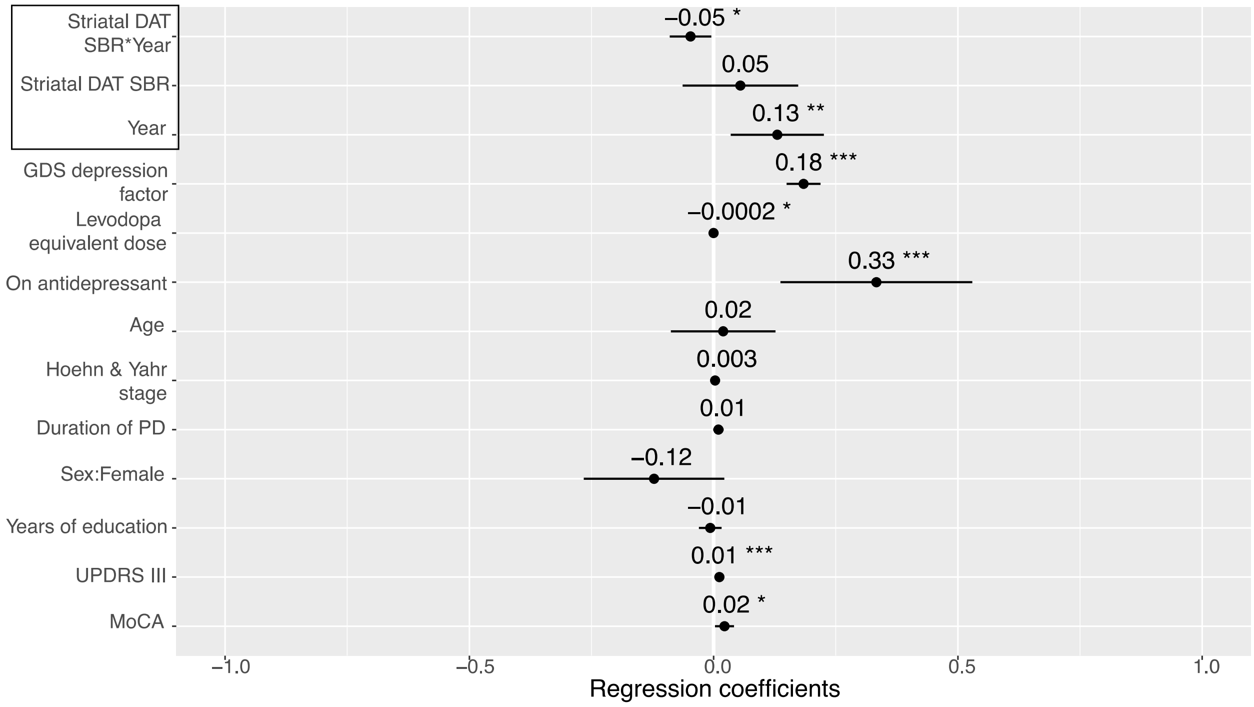


**B.**

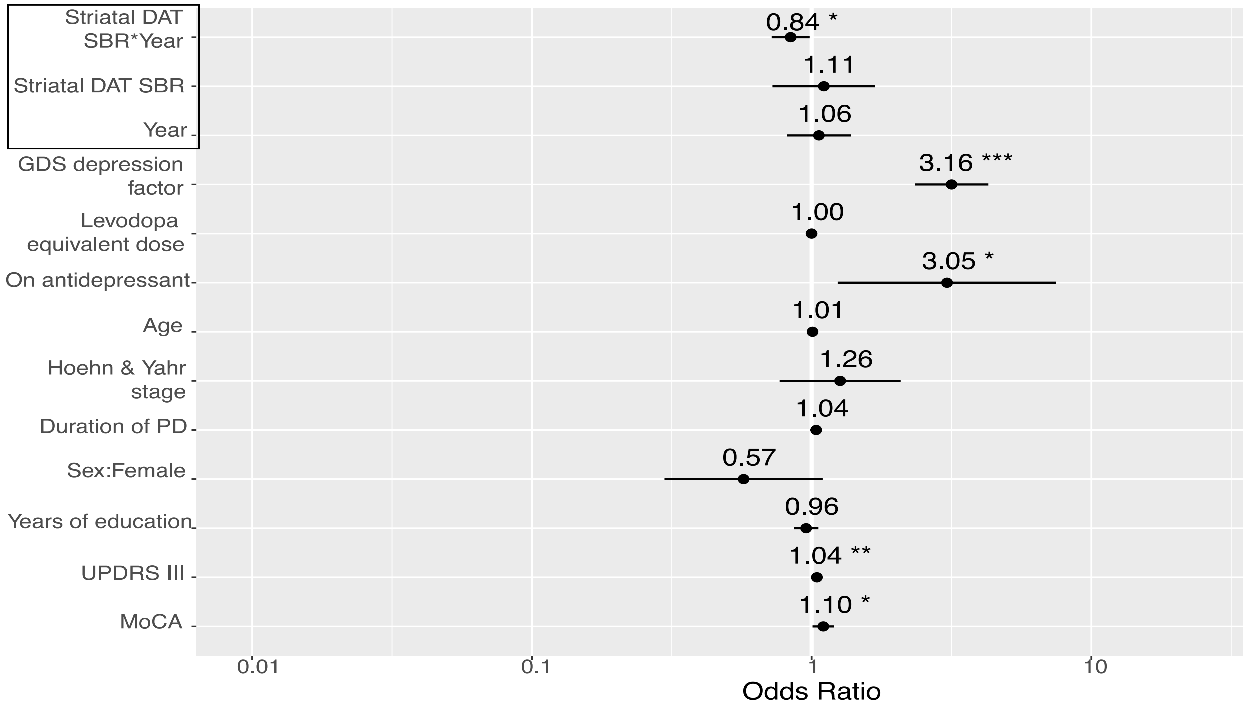


Consistent with our primary analyses, there was a significant interaction between striatal DAT SBR and time (Figure S3A) when treating the GDS-3A as a continuous outcome. Logistic mixed-effects modelling using a categorical GDS-3A dependent variable (score <2 or ≥2, indicating clinically significant apathy), revealed significantly increased odds of clinically relevant apathy with decreasing striatal DAT SBR as time progressed (odds ratio=0.84, 95%CI [0.73 0.98], p=0.031) (Figure S3B).

**Figure S4.** Association between striatal dopamine transporter (DAT) specific binding ratio (SBR) and apathy/anhedonia score using change-from-baseline (BL) SBR values (top) and absolute SBR values (bottom). Individual linear regression models at different years are displayed. All change-from-baseline models were non-significant. Points represent estimated regression coefficients and bars represent 95% confidence intervals; p<0.05*, p<0.01**, p<0.001***.

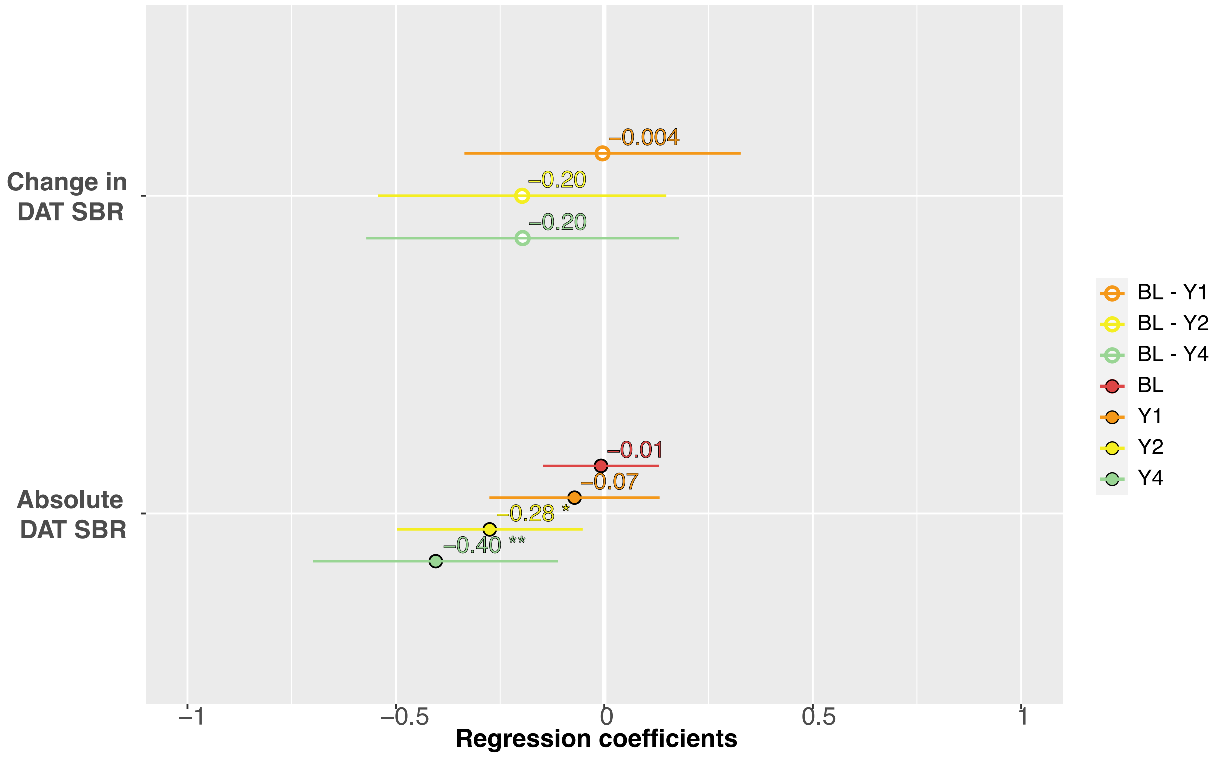

**Figure S5.**

Mixed-effects model investigating the longitudinal relationship between striatal dopamine transporter (DAT) binding ratio (SBR) and apathy/anhedonia at post-baseline assessments, adjusted for baseline striatal DAT SBR (NB this makes the striatal DAT SBR main effect equivalent to change from baseline). Points represent estimated regression coefficients and bars represent 95% confidence intervals; p<0.05*, p<0.01**, p<0.001***.

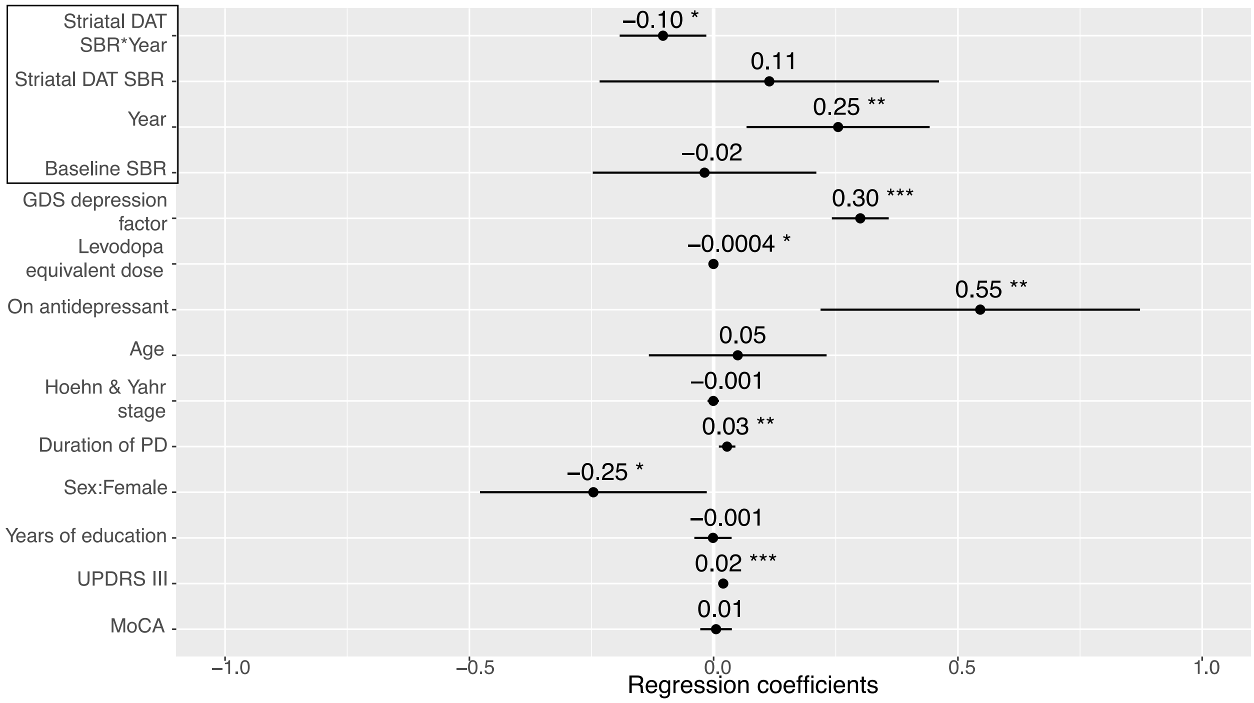


Baseline DAT SBR did not significantly predict subsequent apathy/anhedonia score (β=-0.02, 95%CI [-0.25 0.21], p=0.9), and the interaction between striatal DAT SBR and time remained significant (β=- 0.10, 95%CI [-0.19 -0.01], p=0.023) (Figure S4).
